# TENS/EMG-guided myocentric mandibular positioning: a systematic review and evidence map

**DOI:** 10.3389/froh.2026.1810650

**Published:** 2026-06-23

**Authors:** Sherif Sadek, Mahmood Alamin, Mona Alardah, Diaaeldin Farag

**Affiliations:** 1 Prosthodontic Department, Faculty of Dentistry, Cairo University, Cairo, Egypt; 2 Prosthodontic Division, Restorative Dental Sciences Department, College of Dentistry, Gulf Medical University, Ajman, United Arab Emirates; 3 College of Dentistry, Gulf Medical University, Ajman, United Arab Emirates; 4 Fixed Prosthodontic Department, Faculty of Dentistry, Suez Canal University, Ismalia, Egypt; 5 Restorative Dental Sciences Department, College of Dentistry, Gulf Medical University, Ajman, United Arab Emirates

**Keywords:** EMG, myocentric relation, neuromuscular dentistry, posture, sports dentistry, TMD

## Abstract

**Introduction and aims:**

Jaw registration protocols using TENS and/or EMG have been proposed to identify a physiologically relaxed mandibular position. Occlusal splints, neuromuscular orthoses, and mouthguards fabricated in this position have been proposed to improve masticatory muscle function, body posture, athletic performance, and temporomandibular disorder (TMD) symptoms, but the evidence is limited and heterogeneous. This review aimed to systematically map and appraise the evidence on neuromuscular/myocentric mandibular positioning (TENS- and EMG-guided jaw posture) and its associations with (1) masticatory and postural muscle activity, (2) posture/locomotor symmetry, and (3) TMD-related symptoms and athletic performance.

**Methods:**

This systematic review followed PRISMA 2020 guidelines and was registered in PROSPERO (Registration ID: CRD420251125459). A PICOS framework was used to define eligibility criteria. Human studies assessing neuromuscular/myocentric mandibular position or appliances (neuromuscular orthoses, myocentric splints, neuromuscular mouthguards) and comparing them with conventional occlusion (centric relation, centric occlusion, habitual occlusion, or standard custom-made mouthguards) were included. Primary outcomes were EMG activity, postural/balance measures, and TMD symptoms; secondary outcomes were strength and performance measures. Risk of bias was assessed with RoB 2 for randomized/crossover trials and ROBINS-I for non-randomized studies. Due to clinical and methodological heterogeneity, a narrative synthesis was conducted, and the certainty of the evidence was assessed using the GRADE approach.

**Results:**

Six studies met inclusion criteria (two crossover RCTs, three within-subject experimental studies, and one non-randomized pre–post cohort; total *n* ≈ 493); however, clinical TMD evidence derives from a single non-randomized pre–post cohort (*n* = 313). In healthy/athletic samples, myocentric positioning or appliances were associated with short-term changes in masticatory EMG and small, task-specific differences in balance, running symmetry, and explosive performance outcomes. In the single TMD cohort study, symptom improvement and changes in EMG patterns were reported after 3 months of neuromuscular orthosis wear, but causal inference is limited by a non-randomized pre–post design and potential confounding. Certainty ranged from low to moderate across domains.

**Conclusions:**

Evidence is sparse and methodologically limited; current findings are hypothesis-generating and do not justify routine clinical or performance claims.

**Clinical relevance:**

Neuromuscular/myocentric approaches may be considered in selected cases as an adjunct within DC/TMD-aligned care pathways, but current evidence is insufficient to support routine adoption or performance-related claims.

**Systematic Review Registration:**

https://www.crd.york.ac.uk/PROSPERO/view/CRD420251125459, PROSPERO CRD420251125459.

## Introduction

Temporomandibular disorders (TMD) represent a significant challenge in oral and maxillofacial medicine. A recent large-scale meta-analysis estimated a global prevalence of nearly 34% for TMD signs and symptoms in the general population (with higher rates among females) (1). Additionally, degenerative changes and arthralgia of the temporomandibular joint (TMJ) complex have been reported in up to 17% of specific populations (2) and among certain malocclusion types reached up to 40% (3). Given this burden, approaches that address functional components of occlusion, neuromuscular control, and posture may have both clinical and performance-optimization implications.

Contemporary evidence also supports a biopsychosocial model of TMD, with anxiety, depression, stress, somatization, and pain catastrophizing showing consistent associations with TMD onset, symptom severity, and pain-related disability (4, 5). While neuromuscular approaches primarily target the biomechanical and neuromotor components of TMD, they must be viewed within this broader biopsychosocial context rather than as stand-alone solutions.

The concept of neuromuscular dentistry proposes that the jaw, its musculature, the temporomandibular joint, and associated neural circuits form a “functional unit” whose alignment (termed neuromuscular or myocentric relation) may influence not only TMD symptoms but also postural control and athletic performance (6, 7). The underlying premise is that by de-programming habitual occlusion (e.g., via ULF-TENS) and locating a physiological mandibular posture with minimal muscular strain, one can modulate masticatory muscle activity, reduce joint loading, and thereby impact the broader musculoskeletal chain.

For consistency throughout this review, the term “neuromuscular/myocentric positioning” is used to describe mandibular positions established using TENS-assisted muscle deprogramming and/or EMG-guided recording protocols intended to identify a physiologically relaxed mandibular posture. Where appropriate, the more specific term “TENS/EMG-guided mandibular positioning” is used to emphasize the recording methodology rather than to imply acceptance of a singular or definitive mandibular position concept.

The stomatognathic system and its influence on body posture have been a topic of investigation for over two decades. Studies employing surface electromyography (sEMG) and stabilometric platforms have demonstrated that changes in mandibular position (e.g., the insertion of an acrylic wafer to simulate neuromuscular/myocentric occlusion) can result in statistically significant changes in the EMG activity of cervical, erector spinae, and soleus muscles (8, 9). For example, the study by Bergamini et al. noted a reduction in the mean voltage of neck and postural muscles 15 min after inserting a wafer designed to balance occlusal forces.

Moreover, experimental studies in athletes and semi-athletic populations have similarly investigated whether occlusal interventions (mouthguards or splints) and mandibular positioning can modify jump performance, balance, gait symmetry, or strength outcomes, with some reporting performance gains and others finding minimal or no effect (10). Overall, the literature across both clinical and athletic domains remains fragmented and methodologically heterogeneous: occlusion–posture links are often demonstrated under controlled experimental conditions, but their clinical significance, durability, and impact on functional performance remain uncertain (11).

Despite increasing clinical use and marketing of appliances fabricated after TENS/EMG-guided registration of “neuromuscular” appliances, the evidence base remains difficult to interpret because studies differ in how the myocentric position is established (TENS parameters, EMG configuration, jaw-tracking thresholds) and because outcomes are dispersed across EMG, stabilometry/kinematics, symptoms, and performance measures. To date, there is limited synthesis that specifically isolates TENS-/EMG-guided myocentric registration and simultaneously appraises certainty across these outcome domains. An evidence map with domain-level certainty grading can clarify which outcome domains have signal, which remain equivocal, and where higher-quality comparative trials are most needed (12, 13).

From the TMD perspective, neuromuscular/myocentric occlusion aims to reduce parafunctional masticatory muscle activity, re-establish symmetrical recruitment patterns, and thereby diminish pain, joint noises, and dysfunction. However, comparative evidence supporting clinical effectiveness over established conservative care remains limited (14). From a sports dentistry perspective, improved mandibular posture may facilitate optimal head-neck alignment, enhance neuromuscular coordination, and possibly augment lower-limb or core muscle activation, thereby contributing to performance gains (such as jump height and rate of force development) as observed in some pilot studies.

Accordingly, the objective of this systematic review is to rigorously evaluate existing evidence on the effect of neuromuscular/myocentric relation of the mandible on (i) muscular (masticatory and postural) activity, (ii) postural and balance outcomes, and (iii) clinical symptoms of TMD and/or athletic performance measures. We will focus on both TMD patient populations and athletic/active cohorts, compare neuromuscular/myocentric interventions (splints, wafers, neuromuscular deprogramming) with traditional centric relation/centric occlusion protocols, and assess methodological quality and bias in these studies. The aim is to provide evidence-based guidance for prosthodontic, sports dentistry, and musculoskeletal rehabilitation practices. The central hypothesis was that neuromuscular/myocentric mandibular positioning is associated with measurable differences in muscle activity, postural stability, and clinical or performance outcomes compared with conventional occlusal relations. Given the longstanding debate and variable commercial framing of neuromuscular/myocentric approaches, a conservative synthesis prioritizing study design and certainty is necessary.

Clarifying the functional consequences of mandibular registration is particularly relevant for prosthodontists and restorative dentists, for whom occlusal positioning directly influences appliance design, rehabilitation stability, and patient-reported outcomes.

## Materials and methods

### Study design and registration

This research was conducted as a systematic review, following the methodological and reporting principles outlined in the Preferred Reporting Items for Systematic Reviews and Meta-Analyses (PRISMA 2020) statement and the Cochrane Handbook for Systematic Reviews of Interventions. The review protocol was prospectively registered with the PROSPERO International Prospective Register of Systematic Reviews (Registration ID: CRD420251125459) before data extraction, ensuring transparency and preventing selective reporting. No amendments were made to the review methods after protocol registration.

The primary objective of this review was to systematically identify, evaluate, and synthesize evidence on the effect of neuromuscular/myocentric jaw relation therapy on (1) masticatory and postural muscle activity, (2) body posture and balance, and (3) temporomandibular disorder (TMD) symptoms or functional performance in both athletic and patient populations. The review aimed to bridge two clinical domains, prosthodontic rehabilitation and sports performance, by assessing how neuromuscular/myocentric jaw alignment may influence both symptomatic and physiological neuromuscular function.

### Eligibility criteria

Studies were screened and selected according to the PICOS framework (Population, Intervention, Comparator, Outcomes, Study design).

#### Population

Human participants of any age or sex, including those diagnosed with TMD according to standardized clinical diagnostic criteria (RDC/TMD or DC/TMD), where applicable and healthy individuals or athletes evaluated for performance or postural changes.

#### Intervention

Neuromuscular/myocentric mandibular alignment techniques are obtained through transcutaneous electrical neural stimulation (TENS), surface electromyography (EMG), or computer-assisted jaw tracking to establish a relaxed muscle position. Interventions included the use of neuromuscular orthotics, myocentric splints, or EMG-guided mouthguards fabricated from this recorded jaw position.

#### Comparator

Conventional occlusal positions or appliances, such as centric relation (CR), centric occlusion (CO), leaf gauge, habitual occlusion, or standard custom-fitted mouthguards fabricated using conventional impressions.

#### Outcomes

Primary outcomes included changes in EMG activity of masticatory or postural muscles, alterations in postural stability or balance parameters, and clinical improvements in TMD pain, range of motion, or joint sounds. Secondary outcomes comprised muscular strength, anaerobic power, rate of force development, and functional symmetry in athletes.

#### Study design

Only full-text peer-reviewed studies published in English between 1990 and the 5th of July 2025 were considered, including randomized controlled trials (RCTs), crossover designs, prospective or quasi-experimental clinical studies, and observational studies with a defined comparator. Case reports, reviews, expert opinions, and non-English articles were excluded. Animal and *in-vitro* experiments were also excluded.

### Information sources and search strategy

A comprehensive electronic search was conducted across five databases: PubMed/MEDLINE, Scopus, Web of Science, Cochrane Library, and Google Scholar, from 1990 until 5th of July 2025. The search strategy combined Medical Subject Headings (MeSH) terms and free-text terms related to neuromuscular occlusion, myocentric relationship, temporomandibular disorders, and postural or athletic performance outcomes.

An example of the PubMed search syntax was:

(“neuromuscular occlusion” OR “myocentric relation” OR “myocentric occlusion”) AND (“TMD” OR “temporomandibular disorder” OR “TMJ”) AND (“electromyography” OR “EMG” OR “muscle activity” OR “posture” OR “balance” OR “athletes” OR “performance”).

Filters were applied to include only human studies, those written in English, and those with a clinical or experimental design. The reference lists of relevant articles and reviews were manually searched for additional studies, and grey literature was screened in Google Scholar to capture potentially unindexed research. Full search strategies for all databases are provided in Table 1.

All retrieved records were reviewed, and duplicates were removed before screening.

**Table 1:** Reasons for exclusion after full-text review*.*

Reasons for Exclusion	Details/Subcategories
Inappropriate study design (*n* = 11)	Case reports (*n* = 4) Reviews (*n* = 4) Pilot studies (*n* = 3)
Not addressing the NM approach. (*n* = 4)	TENS used without an Orthotic (*n* = 1) No TENS used before bite registration (*n* = 2) Testing traditional CR rather than myocentric (*n* = 1)
Full text not available (*n* = 2)	Article unavailable in full text through institutional or public sources (*n* = 2)
Non-indexed source (*n* = 2)	Sources not retrievable from standard indexing databases (*n* = 2)

NM, neuromuscular; CR, centric relation; CO, centric occlusion.

### Study selection

Two independent reviewers screened titles and abstracts for relevance. Potentially eligible studies underwent full-text evaluation against the predefined inclusion and exclusion criteria. Any disagreements were resolved through discussion or, when required, by consultation with the third senior reviewer to reach a consensus.

**Figure 1:**
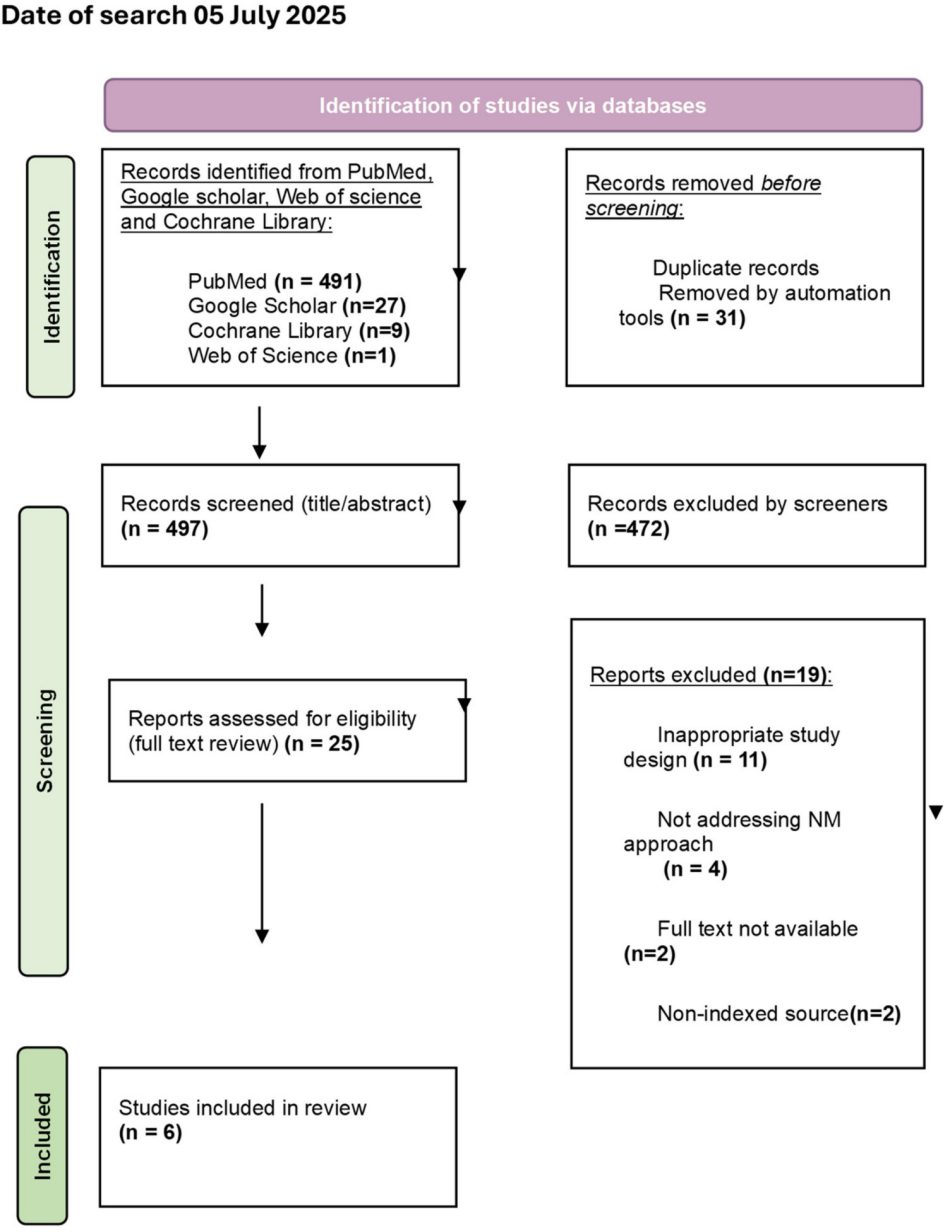
PRISMA flow diagram of study selection process. (Figure 1 illustrates the number of records at each stage of identification, screening, eligibility, and inclusion.).

The study selection process followed PRISMA 2020 recommendations. The study was documented using a PRISMA flow diagram, as shown in Figure 1, which reported the total number of records identified, screened, excluded (with reasons), as shown in Table 1, and ultimately included. Six studies satisfied all inclusion criteria:

- Hickman & Cramer (1998), electromyographic activity in different condylar positions (15).
- Bracco et al. (2004), postural stability analysis across jaw relations (16).
- Cooper & Kleinberg (2008), clinical TMD symptom reduction with neuromuscular orthosis (17).
- Maurer et al. (2015), effect of jaw position on running symmetry (18).
- Maurer et al. (2018), strength and jump performance under varied occlusal splints (19).
- Arent et al. (2010), double-blind crossover trial assessing a neuromuscular dentistry-designed mouthguard on anaerobic power and endurance in athletes (20).

A total of 19 studies were excluded following full-text review for specific methodological or eligibility reasons. Eleven studies were removed due to inappropriate study design, including four narrative or systematic reviews, four case reports, and three pilot studies that lacked adequate control groups or statistical power. These designs were excluded to minimize bias arising from uncontrolled data and to ensure that only studies with sufficient methodological rigor were included. Four studies were excluded because they did not address the neuromuscular (NM) or myocentric approach, including one that used TENS without fabrication of an orthotic, two that performed occlusal adjustment without prior TENS-based deprogramming, and one that assessed traditional centric relation (CR) rather than myocentric positioning. Additionally, two studies were excluded due to a lack of accessible full text through institutional or public databases, and two non-indexed sources were removed because they could not be verified through recognized scientific indexing platforms.

### Data extraction

A standardized data-extraction form was used to ensure consistency across studies. Extracted data included:

- Bibliographic details (author, year, country, journal).
- Study design and sample size.
- Participant characteristics (age, sex, diagnosis, athletic level).
- Description of intervention (TENS/EMG protocols, splint or mouthguard fabrication).
- Comparator condition.
- Primary and secondary outcome measures.
- Main quantitative findings (mean differences, effect sizes, *p*-values).

Data were extracted independently by two reviewers and verified for accuracy. Discrepancies in extraction were uncommon and were resolved by consensus. When essential information was missing, attempts were made to contact study authors for clarification. All extracted data were compiled and organized in Microsoft Excel (Microsoft Corp., Redmond, WA, USA) for descriptive comparison and tabular synthesis before qualitative analysis.

### Risk of bias and quality assessment

The methodological quality of included studies was evaluated using design-appropriate, validated risk-of-bias tools. Randomized and crossover trials (19, 20) were assessed using the Cochrane Risk of Bias 2 (RoB 2) tool. Non-randomized and quasi-experimental studies (15, 16, 17, 18) were evaluated using the ROBINS-I (Risk Of Bias In Non-randomized Studies of Interventions) framework. Two reviewers independently completed the assessments using the structured signaling questions of each tool; discrepancies were resolved through discussion and, when necessary, consultation with a third reviewer.

For RoB 2, domains included bias arising from the randomization process, deviations from intended interventions, missing outcome data, measurement of the outcome, and selection of the reported result. For ROBINS-I, domains included bias due to confounding, participant selection, classification of interventions, deviations from intended interventions, missing data, outcome measurement, and selection of the reported result. Overall judgments followed the guidance specified for each tool.

### Data synthesis and analysis

Because of the heterogeneity across populations, interventions, and outcome measures, a qualitative descriptive synthesis was employed. Quantitative meta-analysis was not appropriate due to variation in EMG units, postural metrics, and performance parameters.

Studies were grouped into five analytical domains: (1) masticatory EMG, (2) posture/balance, (3) locomotion/kinematics, (4) TMD clinical outcomes, and (5) athletic performance.

For each domain, effect direction, magnitude, and statistical significance were summarized, and consistency across studies was qualitatively compared. Where available, we extracted the summary statistics reported by the original study authors (means, standard deviations, and *p*-values), but no standardized effect measures (e.g., standardized mean differences) or pooled estimates could be calculated due to heterogeneity in outcome definitions and reporting. Subgroup interpretation differentiated TMD patient studies from athletic performance trials and acute occlusal adjustments (≤ 1 day) from long-term therapeutic interventions (> 1 week).

Certainty of evidence for each main outcome domain (EMG activity, posture and balance, TMD symptoms, and athletic performance) was assessed independently by two reviewers using the GRADE approach, with consensus reached through discussion. Overall certainty ranged from low to moderate (EMG and performance outcomes) to moderate (posture outcomes) and low (TMD clinical outcomes). Domains considered included risk of bias, inconsistency, indirectness, imprecision, and publication bias. Because each synthesis included fewer than 10 studies, formal assessment of publication bias (e.g., funnel plots) was not feasible (21).

In addition to narrative synthesis, an evidence map was developed to visually and tabularly characterize the distribution of included studies and to identify clusters and gaps. The mapping axes were population (TMD patients vs. athletic/healthy participants) and outcome domain (masticatory EMG, posture/balance, locomotion/kinematics, TMD clinical outcomes, and athletic performance). For each study, we overlaid (i) study design tier (randomized/crossover trial; within-subject experimental study; observational/non-randomized clinical study) and (ii) risk-of-bias tier (RoB 2: low/some concerns/high; ROBINS-I: low/moderate/serious/critical). Outcome direction was qualitatively coded as favoring neuromuscular/myocentric, neutral/no clear difference, or inconsistent/mixed, based on the prespecified primary outcome(s) reported in each study. The evidence map was used alongside domain-level GRADE judgments to support interpretation of certainty and to highlight research priorities.

### Ethical considerations

Ethical approval was not required for this systematic review because it synthesized data from previously published studies and did not involve new data collection from human participants.

## Results

### Study selection

The initial search across PubMed, Scopus, Web of Science, Cochrane Library, and Google Scholar retrieved 528 records. After duplicate removal, 497 articles were screened by title and abstract. Of these, 25 full-text studies were evaluated for eligibility. Nineteen were excluded for reasons such as inappropriate study design (*n* = 11; including four narrative or systematic reviews, four case reports, and three pilot studies), not addressing the neuromuscular/myocentric approach (*n* = 4), lack of accessible full text (*n* = 2), and non-indexed sources (*n* = 2). Ultimately, six studies met the inclusion criteria and were synthesized.

### Characteristics of included studies

Table 2 summarizes the methodological features of the six included investigations, which together comprise a combined sample of 493 participants (313 TMD patients and 180 healthy or athletic subjects). Publication years ranged from 1998 to 2018, representing both foundational and contemporary work.

**Table 2:** Study characteristics table.

Study Name	Author(s)	Publication Year	Journal	Study Design	Sample Size	Population	Ages
Hickman & Cramer (15)	Hickman & Cramer	1998	Oral Surgery, Oral Medicine, Oral Pathology, Oral Radiology	Comparative experimental study	20	Normal adults	Mean age ∼ 26.9 (23−39)
Bracco et al. (15)	Bracco et al.	2004	Neuroscience Letters	Experimental crossover	95	Asymptomatic volunteers	18–52
Cooper & Kleinberg (17)	Cooper & Kleinberg	2008	The Journal of Craniomandibular & Sleep Practice	Clinical trial	313	TMD patients	Mean age ∼ 40
Arent et al. (20)	Arent et al.	2010	Comparative Exercise Physiology	Double-blind crossover RCT	22	Athletes	18–34
Maurer et al. (18)	Maurer et al.	2015	PLoS ONE	Experimental crossover	20	Athletes	∼ 33.9 ± 5.8
Maurer et al. (19)	Maurer et al.	2018	PLoS ONE	Experimental, Randomized crossover trial	23	Athletes	∼ 34.0 ± 10.3

One study investigated neuromuscular orthosis therapy in TMD patients (17), whereas the remaining five experimental or crossover trials were conducted in healthy or athletic populations (15, 16, 18, 19, 20).

All interventions employed TENS- and/or EMG-guided myocentric recordings to establish mandibular position or fabricate orthotics. Comparator conditions included centric relation (CR), centric occlusion (CO), leaf-gauge (LG) positions, habitual bite, or standard custom-fitting mouthguards.

Study designs consisted of two randomized crossover trials (19, 20), three within-subject experimental studies (15, 16, 18), and one prospective longitudinal clinical trial (17). Risk of bias was judged as some concerns for the two crossover randomized trials, primarily due to limitations in blinding procedures and potential selective reporting. For non-randomized and quasi-experimental designs, risk of bias ranged from moderate to serious, driven mainly by confounding, participant selection, and absence of parallel control groups. No study was judged at low risk of bias across all domains. Detailed domain-level assessments are presented and summarized in Table 4.

### Electromyographic activity and neuromuscular function

Two studies specifically assessed surface electromyography (sEMG) outcomes. Hickman and Cramer (15) compared four mandibular registrations, CO, CR, LG, and neuromuscular, in 20 healthy adults. They reported the highest summed EMG activity of the masseter and temporalis muscles during maximal voluntary clenching in the neuromuscular/myocentric (antero-inferior) position, whereas CR produced the lowest activation. This finding indicated that the neuromuscular/myocentric position enabled different patterns of muscle recruitment under load.

In contrast, Cooper and Kleinberg (17) conducted a large-scale prospective study on 313 TMD patients fitted with EMG-verified neuromuscular orthotics, which were worn continuously for three months. At follow-up, patients demonstrated significant reductions in resting EMG activity and improved functional EMG symmetry, along with resolution or marked relief of pain and joint noises, with 80% improvement by the end of the 3-month interval. These findings suggest that neuromuscular/myocentric interventions may influence patterns of masticatory muscle activity, reducing hyperactivity in TMD populations while enhancing functional activation in healthy individuals.

Interpretation is limited by small sample sizes, heterogeneity in EMG acquisition/processing, and the fact that EMG changes are surrogate markers that do not necessarily translate into durable clinical benefit.

### Postural and balance outcomes

Postural stability was evaluated in Bracco et al. (16) and Maurer et al. (18). Bracco and colleagues analyzed 95 asymptomatic adults across three occlusal conditions: CO, rest (cotton-roll position), and myocentric (TENS-guided) position, using a force platform to measure center-of-pressure displacement. They found a statistically significant improvement in frontal-plane postural balance when participants adopted the myocentric position (*p* < 0.05), suggesting an association between mandibular positioning and postural stability measures.

Similarly, Maurer et al. (18) investigated 20 recreational runners fitted with individualized neuromuscular splints. Three-dimensional gait analysis revealed greater left–right symmetry and smoother kinematic patterns during treadmill running with the splint compared with habitual occlusion (*p* < 0.05). Collectively, these studies support the possibility that mandibular positioning may be associated with changes in posture and dynamic balance.

These postural findings largely reflect short-term, within-subject experimental conditions; durability and clinical significance remain uncertain.

### Strength and athletic performance outcomes

Two experimental trials, Arent et al. (20) and Maurer et al. (19), assessed athletic performance variables. Arent et al. (20) conducted a double-blind, randomized, crossover trial in 22 elite male athletes, comparing a neuromuscular dentistry-based mouthguard (Pure Power Mouthguard, PPM) with a standard, custom-fitted mouthguard (CFM). Wearing the PPM significantly improved vertical-jump height (67.6 ± 9.4 cm vs. 65.3 ± 8.6 cm; *p* = 0.003), Wingate peak power (11.6 ± 1.7 vs. 11.1 ± 1.5 W·kg⁻¹; *p* = 0.038), and average power across repeated intervals (*p* < 0.05). No significant differences were noted in bench-press endurance (*p* > 0.48).

Maurer et al. (19) corroborated these findings in 23 athletes using myocentric and centric occlusal splints. They reported 3%–12% increases in jump height, leg press force, and rate of force development compared with habitual occlusion (*p* < 0.05). The authors proposed that improved mandibular–cervical alignment may influence neuromotor coordination and the efficiency of central drive.

Together, these results indicate that neuromuscular alignment may yield modest yet consistent gains in lower-limb power and anaerobic performance, particularly during dynamic lower-body tasks rather than upper-limb endurance tasks.

Given crossover designs, limited blinding in fitting procedures, and narrow athlete demographics, expectancy and context effects cannot be ruled out.

Temporomandibular Disorder Symptoms and Clinical Outcomes

Beyond electromyographic normalization, Cooper and Kleinberg (17) provided signals of symptom improvement. After three months of neuromuscular orthotic therapy, over 80% of patients reported complete or substantial symptom relief, including reductions in myofascial pain, headaches, and TMJ noises. Improvements were accompanied by normalization of EMG profiles and an increased mandibular range of motion. No adverse effects were reported. These findings suggest potential therapeutic benefit, but causal inference is limited because the study was non-randomized and pre-post, and was susceptible to regression to the mean, co-interventions, and expectation effects.

### Overall synthesis

Across the six included studies, findings generally trended in the same direction across outcome domains; however, effects varied by population, intervention protocol, and outcome measure. Given heterogeneity and risk of bias, the body of evidence supports possible associations between neuromuscular/myocentric positioning and selected EMG, postural, symptom, and performance outcomes, but does not permit pooled estimates or definitive causal conclusions. Across domains, the certainty of the evidence ranged from low to moderate, with downgrading primarily due to small sample sizes, nonrandomized designs, indirectness, and methodological heterogeneity, as shown in Tables 3, 4.

**Table 3:** Primary outcomes and key findings.

Study	Design	Population	Intervention	Comparator/Control	Outcomes Assessed	Key Findings
Hickman & Cramer (15)	Within-subject comparative study	20 healthy adults (9F/11M)	CO, CR, LG, and neuromuscular/myocentric registrations with bite platforms constructed for each position	Participants served as their own controls across conditions	sEMG activity of the anterior temporalis and masseter muscles during maximal voluntary clench	Different mandibular positions produced different EMG activation patterns; the neuromuscular/myocentric position showed greater summed clench EMG activity.
Bracco et al. (16)	Experimental crossover study	95 asymptomatic adults (23M/72F), mean age 29 ± 10 years	Three jaw positions evaluated: centric occlusion, rest position (cotton rolls), and TENS-guided myocentric position using K6 jaw tracking	Participants tested under all conditions	Postural sway and stabilometric center-of-pressure measures	The myocentric position was associated with improved frontal-plane postural stability.
Cooper & Kleinberg (17)	Prospective pre–post clinical study	313 TMD patients	Neuromuscular orthosis fabricated using TENS and jaw tracking; worn continuously for approximately 3 months.	Pretreatment baseline	TMD symptoms, resting/function sEMG, mandibular range of motion, TMJ sounds	Reported reductions in pain and joint symptoms with associated changes in EMG patterns and mandibular function.
Arent et al. (20)	Double-blind crossover trial	22 athletes	Neuromuscular/myocentric mouthguard fabricated using a TENS/EMG-guided protocol.	Standard custom-fitted mouthguard	Anaerobic power and muscular endurance	Small improvements in selected anaerobic performance measures were observed with the neuromuscular mouthguard.
Maurer et al. (18)	Repeated-measures crossover study	20 recreational runners	Neuromuscular/myocentric splints, CR splints, MIP splints, and neutral condition	Habitual occlusion and alternative splint conditions	Running symmetry and locomotor kinematics	Neuromuscular/myocentric splints were associated with greater left–right running symmetry and smoother kinematic patterns.
Maurer et al. (19)	Randomized crossover trial	23 athletes	Neuromuscular/myocentric, CR, and MIP splints with neutral condition	Habitual occlusion and alternative splint conditions	Maximal isometric strength, jump height, and rate of force development	Neuromuscular/myocentric splints were associated with modest increases in jump height, leg-press force, and rate of force development.

NM, neuromuscular; CR, centric relation; CO, centric occlusion; MIP, maximum intercuspation.

**Table 4:** Evidence map and certainty by outcome domain.

Outcome domain	Studies (*n*)	Participants	Population (s)	Intervention	Comparator(s)	Overall direction	Key limitations	Risk-of-bias summary	GRADE certainty
Masticatory EMG	2	333	Healthy adults; TMD patients	TENS/EMG-guided myocentric positioning; neuromuscular orthosis	CO/CR/LG; pretreatment baseline	Favors neuromuscular/myocentric positioning	Small samples; heterogeneous EMG protocols; nonrandomized TMD cohort	ROBINS-I: moderate; RoB 2: not applicable	Low–Moderate
Posture/balance	1	95	Asymptomatic adults	TENS-guided myocentric position	CO; rest position	Favors neuromuscular/myocentric positioning	Single short-term study; limited generalizability	ROBINS-I: moderate	Moderate
Locomotion/kinematics	1	20	Runners	Neuromuscular splint	Habitual occlusion; alternative splints	Favors neuromuscular/myocentric positioning	Very small sample; limited washout; sport-specific findings	Some concerns–moderate	Low
TMD clinical outcomes	1	313	TMD patients	Neuromuscular orthosis (∼3 months)	Pretreatment baseline	Favors neuromuscular intervention	Nonrandomized pre–post design; confounding; expectancy effects	ROBINS-I: moderate–serious	Low
Athletic performance	2	45	Athletes	Myocentric mouthguards/splints	Standard mouthguard; habitual occlusion	Small task-specific performance improvements	Small samples; crossover expectancy effects; limited athlete diversity	RoB 2: some concerns	Low–Moderate

### Evidence map

Table 4 summarizes the distribution of included studies by population (TMD vs. athletic/healthy) and outcome domain (EMG, posture/balance, symptoms, performance), and annotates each study-by-study design and risk-of-bias tier.

## Discussion

This systematic review synthesized evidence across EMG physiology, postural control, clinical TMD outcomes, and athletic performance to evaluate whether neuromuscular/myocentric mandibular positioning is associated with measurable differences beyond conventional occlusal positions. The present review should not be interpreted as validation of neuromuscular dentistry as a comprehensive treatment philosophy, but rather as an evaluation of a limited and heterogeneous body of physiological and clinical studies investigating TENS/EMG-guided mandibular positioning. Taken together, the six included studies indicate a coherent directional signal: neuromuscular alignment is associated with (1) different patterns of masticatory muscle recruitment under load, (2) improvements in static/dynamic postural metrics and running symmetry, (3) symptom reductions in TMD cohorts treated with neuromuscular orthoses, and (4) modest performance gains in explosive lower-limb tasks. These domain-specific findings align with contemporary prevalence and diagnostic frameworks, motivating the optimization of function in a large at-risk population. Recent meta-analyses estimate that global TMD prevalence is roughly one-third of adults, underscoring the clinical and socioeconomic relevance of effective occlusal rehabilitation strategies (1). Importantly, these signals arise from a sparse evidence base with predominantly non-randomized or small-crossover designs; therefore, conclusions should be viewed as hypothesis-generating. No included study provides high-certainty evidence of clinical effectiveness.

Importantly, TENS/EMG-guided mandibular registration should not currently be interpreted as a universally validated or definitive method for establishing mandibular position. Contemporary prosthodontic and TMD practice generally considers neuromuscular/myocentric approaches as adjunctive tools that may complement, rather than replace, clinical assessment of occlusion, temporomandibular joint status, and patient-specific functional findings (2, 4, 22). Reduced EMG activity following deprogramming or orthotic therapy does not necessarily indicate optimal occlusal stability, long-term joint adaptation, improved masticatory efficiency, or a superior clinical prognosis. Furthermore, the interpretation of surface EMG findings is inherently limited by factors such as electrode positioning, soft-tissue thickness, crosstalk from adjacent muscles, and interindividual variability (11, 23). These limitations should temper causal interpretation of the observed findings and reinforce the need for cautious clinical application.

The included TMD and athletic studies should be interpreted within distinct clinical and physiological frameworks. In TMD cohorts, neuromuscular interventions were investigated primarily as therapeutic approaches to reduce pain, muscular hyperactivity, and functional dysfunction within broader contemporary TMD management paradigms (2, 4, 22). In contrast, studies involving healthy or athletic participants explored whether transient alterations in mandibular position could influence posture, locomotor symmetry, or neuromuscular performance in otherwise asymptomatic individuals. Although both domains involve modulation of stomatognathic and cervical neuromuscular systems, therapeutic effectiveness in TMD should not be directly extrapolated to ergogenic or performance-related applications. Accordingly, findings from athletic populations should be interpreted as exploratory physiological observations rather than evidence supporting clinical treatment paradigms (13).

### Mechanistic context

From a neuromechanical perspective, ULF-TENS-assisted deprogramming and EMG-guided myocentric registration plausibly reduce tonic hyperactivity, improve muscle length–tension relationships, and reposition the mandible/condyle in a way that alters afferent input to trigeminal and cervico-trigeminal nuclei. Evidence from studies outside our six studies shows that jaw clenching and mandibular position can modulate postural sway, particularly on unstable surfaces, suggesting shared control between the stomatognathic and postural systems. This is consistent with our included findings of improved frontal-plane stability and symmetry during running when neuromuscular/myocentric positions or splints are used (24).

Neurophysiologically, trigeminal afferent input interacts extensively with cervical spinal and vestibular networks involved in postural regulation. Periodontal mechanoreceptors, muscle spindles of the masticatory musculature, and temporomandibular joint receptors contribute sensory input to trigeminal nuclei, which in turn communicate with reticular, vestibular, and cervical motor systems (7, 11, 24, 25). Alterations in mandibular position may therefore influence cervical muscle recruitment, head stabilization, and postural reflex behavior through sensorimotor integration rather than through purely mechanical occlusal effects. Additionally, jaw clenching has been hypothesized to transiently facilitate motor output and neuromuscular activation, which may partially explain the task-specific improvements observed in explosive athletic performance studies (10, 13, 25). Nevertheless, these proposed mechanisms remain incompletely understood and require confirmation through integrated neurophysiological and biomechanical investigations.

### Consistency and directionality across domains

Across domains, the pattern of findings suggests that neuromuscular/myocentric positioning influences masticatory and postural neuromotor control through mechanisms that extend beyond simple changes in occlusal contact. In the EMG domain, the greater recruitment of the masseter and temporalis muscles in the myocentric position likely reflects a more favorable length-tension relationship and reduced protective co-contraction following ULF-TENS deprogramming. Conversely, the reduction in resting EMG and improved bilateral symmetry observed in symptomatic TMD patients is consistent with decreased nociceptive drive and improved neuromuscular organization once habitual parafunctional patterns are interrupted. Together, these findings suggest that neuromuscular/myocentric positioning does not merely redistribute bite force but modulates underlying motor control strategies in both healthy and symptomatic populations (15).

Alternative explanations should be considered. In performance studies, expectancy effects, altered breathing or jaw-clenching behavior, and familiarity across repeated tests may contribute to observed differences. In posture studies, small changes in head position, attention, or stance can influence stabilometric outputs. In the TMD cohort, regression to the mean, co-interventions, and natural symptom fluctuation are plausible contributors. These factors likely inflate apparent effects in the absence of sham controls or parallel comparison groups.

In the postural domain, improvements in frontal-plane stability and locomotor symmetry are best interpreted through the known connectivity of the trigeminal, cervical, and vestibular systems. Changes in mandibular position alter afferent input from periodontal, muscular, and capsular receptors, potentially recalibrating cervical proprioception and influencing whole-body alignment. The observed improvements are therefore unlikely to be purely mechanical; instead, they reflect sensorimotor re-organization that influences mediolateral balance control. Although the absolute magnitude of these changes is modest, the consistent direction across studies suggests a physiologically meaningful interaction between mandibular position and postural regulation (16).

In athletic performance, the ergogenic effects observed in both crossover trials are plausibly explained by enhanced neuromuscular/myocentric coordination arising from improved mandibular–cervical alignment, rather than by changes in occlusal force distribution alone. Altering mandibular position may influence the cranio-cervical complex, reduce parasitic muscle activity, and facilitate more efficient recruitment of lower-limb extensors during explosive movements. However, placebo and expectancy effects cannot be entirely excluded, and the performance benefits appear confined to short-duration, high-intensity tasks rather than endurance or upper-limb activities. These contextual factors must be considered when extrapolating the current findings to broader athletic populations (19, 20).

### Relation to diagnostic standards and clinical practice

Our eligibility framework, aligned with DC/TMD constructs where applicable, allowed outcomes to be interpreted in relation to DC/TMD pain-related and intra-articular constructs and to map onto recognized pain-related and intra-articular domains. This linkage strengthens external validity for prosthodontic and orofacial pain practice (22).

### Strengths and limitations of the evidence

A key strength of the available evidence is the consistent direction of effects across domains, despite substantial methodological diversity. Multiple studies conducted in different populations using various neuromuscular/myocentric protocols reported improvements in at least one outcome related to muscle activity, posture, symptoms, or performance when mandibular position was recorded myocentrically. This convergence suggests that the observed findings may reflect relevant physiological interactions rather than isolated artifacts of individual study designs.

However, several limitations reduce the certainty of the evidence. As outlined in the Methods, the GRADE framework led to domain-specific downgrading, reflecting variation in risk of bias, imprecision, and indirectness. Evidence for EMG outcomes was graded as low to moderate, owing to heterogeneity in sEMG acquisition (electrode placement, filtering parameters) and limited sample sizes. Certainty for posture and balance outcomes was moderate, supported by consistent within-subject findings but constrained by short-term measurement windows and variable stabilometric protocols. Evidence for TMD clinical improvement was graded as low certainty, driven by reliance on a single nonrandomized pre–post cohort with a serious risk of confounding. Finally, athletic performance outcomes were rated with low-to-moderate certainty due to small sample sizes, potential placebo or expectancy effects in crossover designs, and limited inclusion of female athletes and diverse sports.

Additional uncertainty relates to the reproducibility of TENS-guided mandibular positioning. Previous studies have reported variability in the reproducibility of post-TENS mandibular positions across recording sessions and operators, potentially influenced by differences in muscle responsiveness, patient adaptation, deprogramming duration, and recording methodologies. These factors may contribute to variability in neuromuscular registration outcomes and complicate the interpretation of treatment effects (6, 11).

These methodological limitations, combined with diversity in TENS parameters, jaw-tracking instrumentation, splint fabrication methods, and outcome definitions, preclude meta-analysis and limit the precision and generalizability of effect estimates. Moreover, because fewer than ten studies contributed to each outcome domain, formal assessment of publication bias was not feasible. Collectively, these factors indicate that although the overall direction of evidence favors neuromuscular/myocentric positioning, the magnitude and clinical applicability of these effects should be interpreted with caution. Moreover, the long-term stability of TENS/EMG-guided mandibular positions and their relationship to durable occlusal adaptation or clinical prognosis remains insufficiently established.

An additional limitation is that the evidence base was dominated by short-term physiological studies and a single nonrandomized clinical cohort, which limits conclusions regarding long-term therapeutic effectiveness, occlusal stability, and the durability of neuromuscular/myocentric positioning over time.

### Implications for future research

Future work should prioritize methodological standardization to improve comparability and enable quantitative synthesis. Core aspects of neuromuscular/myocentric protocols, including ULF-TENS waveform and duration, EMG electrode configuration, jaw-tracking thresholds, and splint fabrication procedures, should be reported with sufficient detail to satisfy CONSORT and STRICTA-style transparency. High-quality multicenter randomized controlled trials in well-characterized DC/TMD cohorts are particularly needed to compare myocentric appliances with conventional CR-based interventions using masked outcome assessment.

Mechanistic research combining EMG with cervical proprioception testing, stabilometric, and full-body kinematics would help clarify the pathways linking mandibular position to postural and muscular outcomes. In the athletic domain, future trials should incorporate preregistration, sham-guard controls, adequate washout periods, and inclusion of female athletes and diverse sports disciplines to address current gaps.

Establishing standardized core outcome sets that encompass pain intensity, jaw function, EMG symmetry, center-of-pressure metrics, countermovement jump height, and rate of force development will facilitate meta-analyses and accelerate comparative effectiveness research. Advancing these methodological priorities is essential for determining the clinical and performance relevance of neuromuscular/myocentric positioning and guiding its appropriate integration into prosthodontic and sports dentistry practice.

## Conclusion

Across six studies, neuromuscular/myocentric mandibular positioning was variably associated with physiological and functional signals, including more efficient masticatory EMG activation in healthy individuals, improved static and dynamic postural control, substantial symptom reduction in patients with TMD, and modest enhancements in explosive lower-limb performance in athletic populations. While the overall certainty of the evidence ranged from low to moderate across domains, the directionality of the findings was consistent across diverse methodologies and populations.

Because heterogeneity in TENS parameters, EMG acquisition protocols, splint fabrication methods, and outcome metrics limits quantitative pooling, the magnitude of these effects should be interpreted conservatively. Nevertheless, the biological plausibility of trigemino-cervical integration, combined with consistent within-subject improvements, warrants further investigation of the potential role of myocentric recording and neuromuscular orthoses as part of a broader evidence-based approach to prosthodontic rehabilitation and sports dentistry.

Future progress requires high-quality, protocol-standardized randomized controlled trials to refine target populations, quantify true effect sizes, and optimize deprogramming parameters and appliance design. Until such evidence emerges, neuromuscular/myocentric approaches should be considered promising but adjunctive tools rather than definitive replacements for established occlusal and TMD management protocols.

### Clinical relevance

The findings of this review suggest that neuromuscular/myocentric mandibular registration may have potential exploratory applications in selected contexts across both prosthodontic and sports-related contexts, provided that its limitations are acknowledged. For prosthodontists and TMD clinicians, neuromuscular/myocentric techniques may be explored on a case-selective basis in patients with persistent muscular hyperactivity or functional symptoms not fully addressed by conventional conservative management, although current evidence remains insufficient to support routine implementation. Evidence for posture-related outcomes appeared more consistent than evidence for TMD clinical outcomes, although certainty remained limited across domains.

In sports dentistry, available data suggest that neuromuscular/myocentric mouthguards are performance-neutral or may yield small, task-specific improvements, particularly in tasks requiring explosive lower-limb output. Given the low-to-moderate certainty of evidence, such appliances may be integrated into sports programs where mouthguards are already mandatory, but clinicians should avoid overextending claims beyond what current evidence supports. Across both domains, practitioners should aim to standardize ULF-TENS and EMG protocols, document jaw-tracking parameters transparently and prioritize individualized assessment to ensure that any perceived benefits are grounded in physiological rather than expectancy-based effects.

These findings should be interpreted as complementary to, not competing with, established occlusal and DC/TMD-aligned conservative management frameworks.

## Data Availability

The original contributions presented in the study are included in the article/Supplementary Material, further inquiries can be directed to the corresponding author.
